# Immunoreactivity of the AAA+ chaperone ClpB from *Leptospira interrogans* with sera from *Leptospira*-infected animals

**DOI:** 10.1186/s12866-016-0774-8

**Published:** 2016-07-16

**Authors:** Joanna Krajewska, Zbigniew Arent, Daniel Więckowski, Michal Zolkiewski, Sabina Kędzierska-Mieszkowska

**Affiliations:** Department of General and Medical Biochemistry, University of Gdansk, Faculty of Biology, 80-308 Gdańsk, Poland; University Centre of Veterinary Medicine JU-UAK, University of Agriculture in Krakow, 30-059 Krakow, Poland; Departament of Biochemistry and Molecular Biophysics, Kansas State University, Manhattan, KS 66506 USA

**Keywords:** ClpB, *Leptospira interrogans*, Leptospirosis, Molecular chaperone, Pathogen

## Abstract

**Background:**

*Leptospira interrogans* is a spirochaete responsible for leptospirosis in mammals. The molecular mechanisms of the *Leptospira* virulence remain mostly unknown. Recently, it has been demonstrated that *L. interrogans* ClpB (ClpB_Li_) is essential for bacterial survival under stressful conditions and also during infection. The aim of this study was to provide further insight into the role of ClpB in *L. interrogans* and answer the question whether ClpB_Li_ as a potential virulence factor may be a target of the humoral immune response during leptospiral infections in mammals.

**Results:**

ClpB_Li_ consists of 860 amino acid residues with a predicted molecular mass of 96.3 kDa and shows multi-domain organization similar to that of the well-characterized ClpB from *Escherichia coli*. The amino acid sequence identity between ClpB_Li_ and *E. coli* ClpB is 52 %. The coding sequence of the *clpB*_*Li*_ gene was cloned and expressed in *E. coli* BL21(DE3) strain. Immunoreactivity of the recombinant ClpB_Li_ protein was assessed with the sera collected from *Leptospira*-infected animals and uninfected healthy controls. Western blotting and ELISA analysis demonstrated that ClpB_Li_ activates the host immune system, as evidenced by an increased level of antibodies against ClpB_Li_ in the sera from infected animals, as compared to the control group. Additionally, ClpB_Li_ was found in kidney tissues of *Leptospira-*infected hamsters.

**Conclusions:**

ClpB_Li_ is both synthesized and immunogenic during the infectious process, further supporting its involvement in the pathogenicity of *Leptospira*. In addition, the immunological properties of ClpB_Li_ point to its potential value as a diagnostic antigen for the detection of leptospirosis.

## Background

*Leptospira interrogans* belongs to pathogenic spirochaetes causing a serious disease in both humans and animals known as leptospirosis that is considered the most widespread zoonosis of worldwide importance [1]. The vectors of this pathogen are mostly wild rodents and domestic animals, which harbor the spirochetes in the proximal renal tubules of the kidneys and chronically excrete the leptospires with urine into the environment [2]. It is worth noting that leptospirosis is also a serious economic problem, because it causes abortions, stillbirths, infertility, failure to thrive, reduced milk production, and death in domestic animals such as cows, pigs, sheep, goats, horses and dogs [3–6]. In humans the disease varies from an asymptomatic flu-like illness to an acute life-threatening infection. Despite its severity and global importance, the molecular mechanisms of leptospiral pathogenesis remain largely unknown [1]. To date, only a few proteins have been identified as potential virulence factors in *Leptospira*. Among them, there is the chaperone ClpB, a member the Hsp100/Clp subfamily of the AAA+ ATPases that reactivates stress-aggregated proteins in cooperation with the DnaK system [7]. Recently, ClpB from *L. interrogans* (ClpB_Li_) has been shown to be essential for bacterial survival under stressful conditions (nutrient restriction, oxidative and heat stresses) and also for the pathogen’s virulence [8]. The involvement of ClpB in the response of *L. interrogans* to oxidative stress suggests that this chaperone may be one of key mediators of stress resistance, which is a prerequisite for *Leptospira* pathogenesis. The present study provides further insight into the role of ClpB_Li_ during the infectious process. It is known that heat shock proteins (Hsps) play important roles during bacterial infections. They help pathogens to overcome stressful conditions to which they are exposed within the host cells, and represent major targets of the host’s immune system. Taking into account the fact that the chaperone ClpB from some pathogenic bacteria, *Francisella tularensis* and *Mycoplasma pneumoniae*, has been shown to be immunoreactive [9, 10], we decided to investigate an immunogenic potential of ClpB_Li_, which could point to this chaperone’s role in the pathogenicity of *Leptospira* and may translate into diagnostic applications.

## Methods

### Serum samples

We studied archived serum samples from rabbits and cattle. Rabbit antisera (*n* = 8) against *L. interrogans* serovars: Icterohaemorrhagiae, Hardjo, and Canicola, and *L. borgpetersenii* serovars: Hardjo, Javanica, were prepared as described by [11]. Polyclonal rabbit antiserum prepared against the *L. interrogans* ClpB (residues 158–334; anti-ClpB_Li158–334_ serum) [8] and provided by M. Picardeau was used as a positive control and the pre-immune serum was used as a negative control. Bovine sera were collected from cattle (*n* = 10) experimentally infected with *L. borgpetersenii* serovar Hardjo via conjunctival instillation of 1 x 10^6^ bacteria. Blood samples were collected 28 days after the challenge and in one case 210 days after the challenge (this serum was used as a positive control showing the highest OD in ELISA). Sera from uninfected cattle (*n* = 8) and also a fetal bovine serum were used as negative controls. To confirm the serological status of leptospiral infection, the sera were subjected to the microscopic agglutination test (MAT) [11, 12] and used at dilutions 1:100 for Western blotting or 1:200 for ELISA.

### Kidney homogenate preparation

For detection of ClpB_Li_ in kidney tissues from *Leptospira*-infected hamsters, the kidneys were macerated with nine parts of a 1 % BSA diluent and inoculated into Tween80/40/ LH semi-solid medium. Cultures were incubated at 28–30 °C, for up to 10 weeks and examined weekly by dark-field microscopy to detect the growth of leptospires. The same macerated kidney tissues (20 μg sample of homogenate) were used for Western-blotting analysis. Total protein concentration in the homogenates was determined by the method of Bradford [13].

### Plasmid construction for protein overproduction

*L. interrogans clpB* gene (2583 bp) was amplified from genomic DNA of *L. interrogans* by PCR using AccuTaq LA polymerase MIX (Sigma) with the following primers: CATATGAAATTAGATAAACTTACATCCAAATT with the NdeI restriction site underlined, and AAGCTTTTAAACTACAACAACTACC with the HindIII restriction site underlined. DNA primers were synthesized by Genomed S.A. (Warsaw, Poland). First, the PCR product was cloned into pJET1.2 blunt vector (Fermentas), then digested with NdeI, HindIII, and ligated with the linearized pET NdeI-HindIII vector. The sequence of the resulting construct was confirmed by DNA sequencing (Genomed S.A.). *Leptospira* genomic DNA was extracted with a QIAamp DNA Mini Kit (Qiagen).

DNA plasmid preparation and transformation of *E. coli* cells were done according to [14].

### Purification of the recombinant ClpB_L_

*L. interrogans* ClpB protein was overproduced in *E. coli* BL21(DE3) strain (Novagen) and purified according to the procedure similar to that used to obtain ClpB from *Ehrlichia chaffeensis* [15]. Briefly, bacteria were grown at 37 °C to OD_600_ = 0.6 and then induced with 0.5 mM IPTG for 2 h. Next, the cells were collected and suspended in 50 mM Tris–HCl (pH 7.4), 300 mM NaCl, 20 mM imidazole and 0.1 % Triton X-100, then disrupted by sonication in the presence of the protease inhibitor PMSF and centrifuged to collect the soluble extract. Next, polyethyleneimine (PEI) was added to precipitate nucleic acids. After centrifugation (20 000 g, 1 h), the supernatant was applied to a Ni-NTA column (Qiagen) and the bound protein was eluted with 50 mM Tris–HCl (pH 7.4), 300 mM NaCl, and 0.1 % Triton X-100 and 250 mM imidazole. Fractions containing a 6His-tagged ClpB_Li_ (a calculated molecular mass of 98 488.59 Da) were identified with SDS-PAGE electrophoresis and Coomassie blue staining, then combined and further purified by gel filtration on Superdex 200 (Sigma) equilibrated with 50 mM Tris–HCl (pH 7.5), 10 % glycerol, 1 mM EDTA and 1 mM DTT. The pooled fractions containing ClpB_Li_ were dialyzed against dialysis buffer (50 mM Tris–HCl pH 7.5, 1 mM EDTA, 1 mM DTT, 20 mM MgCl_2_, 200 mM KCl, 10 % glycerol) and stored at −70 °C. The N-terminal histidine tag was removed by proteolytic digestion using the Thrombin Cleavage Capture Kit (Novagen) according to the manufacturer’s protocol. The identity of the purified ClpB_Li_ was confirmed by a liquid chromatography-tandem mass spectrometry (LC-MS/MS) analysis of tryptic peptides obtained after trypsin cleavage of the protein, performed at the MS LAB IBB PAN (Warsaw, Poland). The equipment used was sponsored in part by the Centre for Preclinical Research and Technology (CePT), a project co-sponsored by European Regional Development Fund and Innovative Economy, The National Cohesion Strategy of Poland.

### SDS-PAGE and Western blotting analysis

To assess immune reactivity of ClpB_Li_, SDS-PAGE electrophoresis was performed according to [16] using 10 % polyacrylamide gels and Western blotting was performed as described [17]. The blots were blocked with 0.1 % Tween 20 in Tris-buffered saline (TBS) for 1 h at room temperature and then incubated overnight at 4 °C with anti-ClpB_Li158–334_ serum (1: 2000 dilution) [8] or polyclonal rabbit and bovine sera (1:100 dilution) against *Leptospira* strains. After primary antibody incubation, the blots were washed three times with TBS containing 0.05 % Tween 20 and incubated for 1 h at room temperature with the goat anti-rabbit IgG horseradish peroxidase (HRP) conjugate (Sigma) diluted 1: 3000 or the polyclonal rabbit anti-cow Ig/HRP conjugate (DakoCytomation) diluted 1: 1000. The blots were then washed three times as described above and were developed using 3,3′-diaminobenzidine (Sigma), and H_2_O_2_ as substrates.

### ELISA procedure

ELISA (enzyme-linked immunosorbent assay), was performed to analyze the immune response in animals experimentally exposed to *L. interrogans* serovars. Costar 96 well EIA*/*RIA polystyrene high-binding plates were coated with 100 μl of 0.625 μg/ml of the recombinant ClpB_Li_ (a capture antigen) resuspended in phosphate-buffered saline (PBS) by incubation overnight at 4 °C. The plates were then washed five times with PBST buffer (PBS containing 0.05 % Tween 20) and non-specific binding sites were blocked by incubation with 100 μl of 0.1 % Tween 20 in PBS buffer for 1 h at room temperature. The wells were washed five times with PBST buffer. Control and duplicate animal serum samples were diluted 200-fold in PBST buffer and 50 μl of the diluted sera (in duplicate) were applied to each well and incubated at 37 ° C for 1 h, followed by five rinses with PBST buffer. Next, secondary HRP-conjugated anti-rabbit (Abcam) (diluted 1:10 000) or anti-cow IgG (DakoCytomation) (diluted 1:2000) were added to each well and incubated for 1 h at 37 °C. The plates were then washed five times with PBS buffer and 3,3′,5,5′-tetramethylbenzidine (TMB) (Sigma-Aldrich) was added to detect the antibodies. The reaction was stopped after 10 min by the addition of 50 μl of 1 M H_2_SO_4._ The absorbance at 450 nm was measured using PerkinElmer Multimode Plate Reader (Enspire). The assay was performed three times for each serum.

### Data analysis

The statistical significance of differences between the ELISA results obtained for sera collected from uninfected and infected animals were determined using the Welch’s adjusted one-way ANOVA followed by the post-hoc Scheffe multiple comparison test. *P <* 0.05 was considered statistically significant. Results of data analysis are presented in the graphs as the median values. All statistical analyses were performed using STATISTICA PL program.

## Results

### Analysis of the amino-acid sequence of the molecular chaperone ClpB from *L. interrogans*

**Fig. 1:**
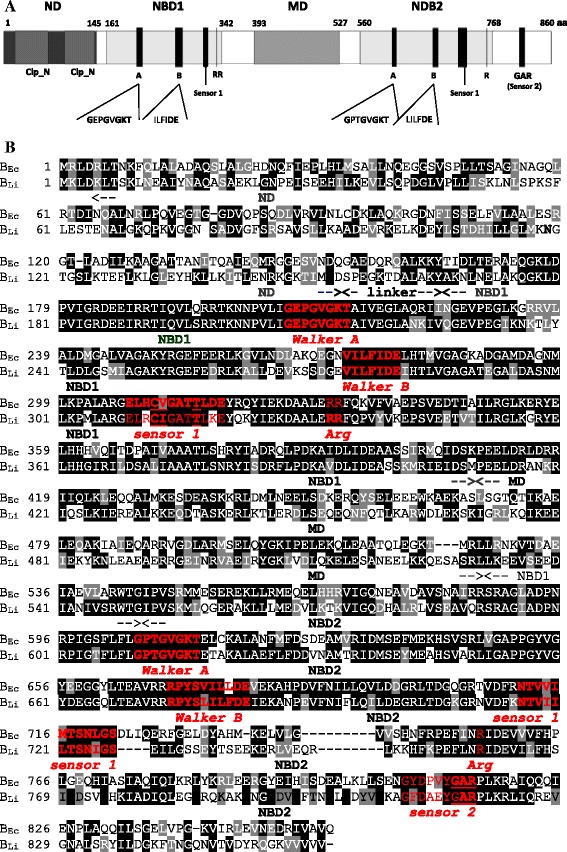
Proposed domain organization of ClpB from *L. interrogans.* **a** The diagram shows structural domains of the protein: N-terminal domain (ND) with the double Clp_N motif, nucleotide binding domain 1 (NBD1), middle coiled-coil domain (MD) and nucleotide binding domain 2 (NBD2). Conserved ATPase motifs such as the Walker A (A), Walker B (B), sensor 1, sensor 2 (GAR) and the Arg fingers (R), coordinating ATP binding and hydrolysis are also indicated. Conserved residues of these motifs are marked in bold. **b** Sequence alignment of ClpB from *E. coli* (B_Ec_) and *L. interrogans* (B_Li_). Domain boundaries are indicated below the amino acid sequence. The conserved motifs are shown in red. Identical and similar amino acid resides are shaded in black and gray, ... respectively

The *clpB*_*Li*_ gene encodes a protein of 860 amino acid residues with a predicted molecular mass of 96325.2 Da. Sequence alignment of ClpB_Li_ (Fig. 1) revealed that this protein shows a multi-domain organization similar to that of the well-characterized ClpB from *Escherichia coli* (ClpB_Ec_). Thus, ClpB_Li_ contains an N-terminal domain (ND_1-145aa_), two nucleotide binding domains (NBD1_161-342aa_, NBD2_560-768aa_) and a middle coiled-coil domain (MD_393-527aa_) (Fig. 1). Both NBDs, involved in ATP binding and ATP hydrolysis, contain all characteristic and conserved sequence mofits of AAA+ ATPases (ATPases *a*ssociated with a variety of cellular *a*ctivities), i.e. Walker A (GX_4_GKT/S), Walker B (Hy_2_DE) and sensor 1/2 motifs. Conserved arginine residues called Arg fingers are also present in both NBD domains. Sequence alignment of the ClpB sequences from bacteria *L. interrogans* and *E. coli* using the Clustal software revealed that the total sequence identity between them is only 52 %; 27.7 % within ND, 45.3 % within MD, 72 % within NBD1, and 65.7 % within NBD2. Therefore, the most highly conserved are the NBD domains and the main differences between *L. interrogans* and *E. coli* ClpB are in the N-terminal domain and the coiled-coil middle domain.

### Expression of the *clpB*_*Li*_ gene in *E. coli cells* and purification of ClpB_Li_

**Fig. 2:**
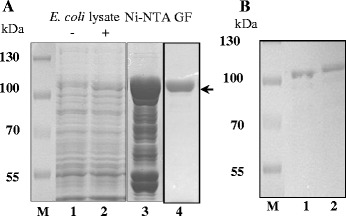
Purification of *L. interrogans* ClpB. **a** The Coomassie blue-stained SDS-PAGE gel showing the lysates from *E. coli* cells transformed with the recombinant plasmid expressing *clpB*_*Li*_ (pET28*clpB*_*L*i_) without induction (−) (lane 1) and induced with IPTG (+) (lane 2), and the representative fractions obtained following the nickel resin (Ni-NTA, lane 3) and gel filtration (GF, lane 4) purification of ClpB_Li_. The arrow indicates the position of the 6His-tagged ClpB_Li_ (~98.5 kDa). **b** The Coomassie blue-stained SDS-PAGE gel showing ClpB_Li_ digested with thrombin (lane 1) and the 6His-tagged ClpB_Li_ (lane 2). The positions of protein size markers (M) (in kDa), PageRuler prestained Protein Ladder (Thermo Scientific), are shown on the ... left

**Fig. 3:**
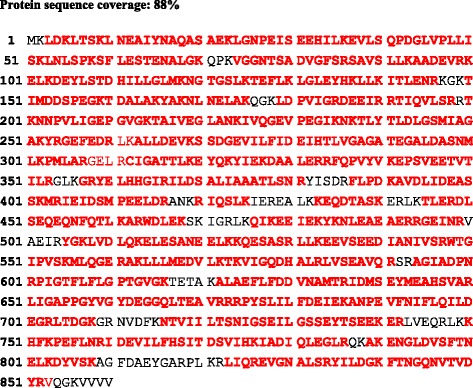
LC-MS/MS analysis of the purified ClpB_Li_. The amino acid sequence of ClpB_Li_ is shown with the peptides detected by LC-MS/MS indicated in ... red

To examine whether ClpB_Li_ shows an immunogenic potential, which could point to its participation in the pathogenicity of *Leptospira,* we obtained a construct expressing *clpB*_*Li*_ (pET28*clpB*_*Li*_) and then overproduced the recombinant ClpB_Li_ as a 6-histidine-tagged protein in *E. coli* B21(DE3) cells. As expected, the expression of pET28*clpB*_*Li*_ resulted in the ~100-kDa protein, corresponding to ClpB_Li_ that was soluble in *E. coli* cells. The protein was purified from the soluble fraction using two separation techniques: immobilized metal affinity chromatography (IMAC) and gel filtration chromatography (Fig. 2a). The identity of ClpB_Li_ was confirmed with an LC-MS/MS analysis (Fig. 3). The obtained peptide map covered 88 % of the amino acid sequence of ClpB_Li_. In addition, LC-MS/MS data indicated that the purified ClpB_Li_ was not contaminated with ClpB from the *E. coli* host strain. The purified ClpB_Li_ was subsequently digested with thrombin to remove the N-terminal 6His-tag (Fig. 2b). The post-cleavage N-terminal sequence of the recombinant ClpB_Li_ protein contains three additional amino acid residues, namely GlySerHis, and in such form the protein was further characterized by Western blotting analysis and ELISA assay.

### Immunogenic capacity of ClpB_Li_

**Fig. 4:**
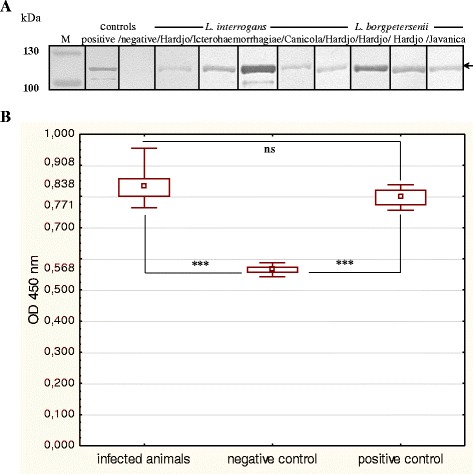
Immune reactivity of the recombinant ClpB_Li_ with rabbit sera. **a** The purified ClpB_Li_ protein (250 ng) was resolved by SDS-PAGE and analyzed by Western blotting using: the antiserum against ClpB_Li158–334_ (a positive control), pre-immune control serum (a negative control), or polyclonal rabbit antisera raised against: *L. interrogans* and *L. borgpetersenii* serovars as indicated in the figure. The positions of protein size markers (M) (in kDa), PageRuler prestained Protein Ladder (Thermo Scientific), are shown on the left. The arrow indicates the position of ClpB_Li_. (**b**) ELISA analysis of the recombinant ClpB_Li_ protein as a capture antigen using all the above rabbit sera. The data were analyzed using Welch adjusted one-way ANOVA. Symbols: (▫), the median value; (box), 25 %–75 % range around the median value; (whiskers), min-max range. (***) denotes *P <* 0.001; ns, not statistically significant

**Fig. 5:**
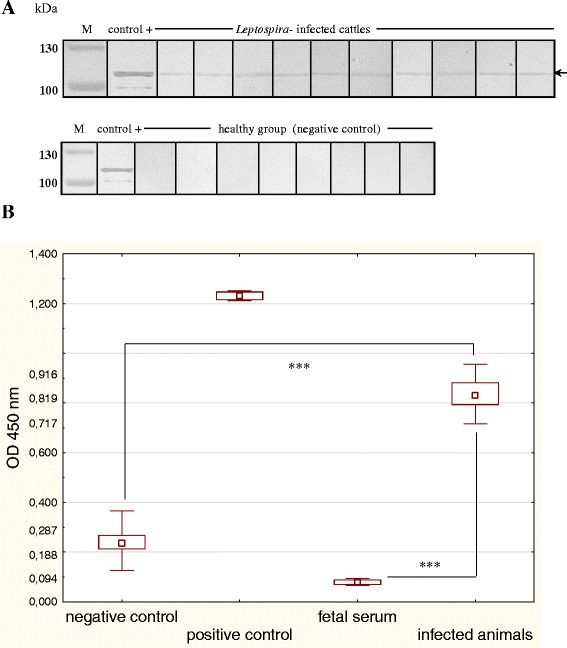
Immune reactivity of the recombinant ClpB_Li_ with bovine sera. **a** The purified ClpB_Li_ protein (250 ng) was resolved by SDS-PAGE and analyzed by Western blotting using the antiserum against ClpB_Li158–334_ (a positive control; control +), polyclonal bovine antisera raised against: *L. borgpetersenii* serovar Hardjo (*Leptospira-*infected cattle), and sera collected from uninfected cattle (healthy group; negative control). The positions of protein size markers (M) (in kDa), PageRuler prestained Protein (Thermo Scientific), are shown on the left. The arrow indicates the position of ClpB_Li_ (~100-kDa). **b** ELISA analysis of the recombinant ClpB_Li_ protein as a capture antigen using the above bovine sera. Fetal bovine serum was also used. The data were analyzed using Welch adjusted one-way ANOVA. Symbols: (▫), the median value; (box), 25 %–75 % range around the median value, (whiskers), min-max range. (***) denotes *P <*... 0.001

The immune reactivity of ClpB_Li_ with serologically positive sera from rabbits and cattle experimentally infected with two pathogenic *Leptospira* species (*L. interrogans* and *L. borgpetersenii*) was tested by Western blotting (Figs. 4a and 5a) and ELISA assay (Figs. 4b and 5b) and compared to the sera from uninfected healthy controls. We found that all the tested sera prepared from *Leptospira*-infected animals, but not from the uninfected controls, strongly reacted with ClpB_Li_ in Western blotting (Figs. 4a, 5a). The ELISA signals of the sera from infected animals were also significantly higher than those of uninfected animals (Figs. 4b, 5b; *P* < 0.001). These results show that *Leptospira* infection induces production of anti-ClpB_Li_ antibodies in animal models. The cross-reactivity between *L. interrogans* and *L. borgpetersenii* is not surprising due to ~95 % sequence similarity between ClpB from those two species [8].

### Detection of ClpB_Li_ in *Leptospira*-infected animals

**Fig. 6:**
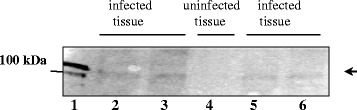
Detection of ClpB_Li_ in hamster kidney tissues. The macerated kidney tissues containing ~20 μg proteins were subjected to SDS-PAGE followed by Western blotting with anti-ClpB_Li158–334_ serum. Lane 1, purified ClpB_Li_ (a positive control); lanes 2, 3, 5, 6, the kidney homogenates from hamsters infected with *L. interrogans* serovar Hardjo and euthanized 14 (lane 2) or 6 (lanes: 3,5,6) weeks post infection; lane 4, the kidney homogenate from an uninfected hamster. The arrow indicates the position of the 6His-tagged ClpB_Li_ (~98.5 kDa). The position of 100-kDa protein marker (PageRuler unstained Protein Ladder; Thermo Scientific), is shown on the ... left

Additionally, we detected ClpB_Li_ (96-kDa protein) in the infected hamster kidney tissue (Fig. 6), from which leptospires were isolated using standard culture method. No reactivity of the 96-kDa protein with anti-ClpB_Li158–334_ serum was observed in the kidney homogenate obtained from an uninfected hamster (Fig. 6, lane 4). The result indicates that ClpB_Li,_ is produced during an experimental infection of animals.

In summary, our data indicate that the molecular chaperone ClpB_Li_ is immunogenic and detectable in animals infected with pathogenic *Leptospira* spp.

## Discussion

Leptospires like many other pathogenic bacteria are exposed to a significant stress during infection of host cells, frequently resulting in protein misfolding and aggregation. Despite being exposed to stressful conditions, pathogens survive, overcome host defense mechanisms, and cause the disease symptoms. The specific mechanisms of the host invasion by leptospires are not well defined. In particular, the molecular basis for virulence remains unknown, due to the lack of genetic tools for the manipulation of *Leptospira*. The fact that ClpB is usually up-regulated in pathogenic microorganisms [8, 15] suggests that the disaggregase activity of ClpB may be essential for their virulence. Moreover, the involvement of ClpB in the response of *L. interrogans* to oxidative stress [8] suggests that this chaperone may be one of key mediators of stress resistance, which is a prerequisite for *Leptospira* pathogenesis. The chaperone ClpB may function either as a true virulence factor directly involved in causing the disease or a virulence-associated protein that can be essential for colonization of the host. Virulence gene products are often immunogenic and responsible for acquired immunity that protects against disease [18]. At this point it should be also noted that molecular chaperones despite their cytosolic localization are strongly immunogenic in a number of bacterial infections [19]. It has been reported that some chaperones (e.g. GroEL) may be associated with the outer membrane of the pathogenic bacteria or exported from the bacterial cell after heat shock [19]. Therefore, exposure of bacterial Hsps to the host’s immune system is possible during infection. Indeed, ClpB from some pathogens (i.e. *Mycoplasma pneumoniae*, *Francisella tularensis*) is an immunoreactive protein [9, 10]. The total sequence identity between ClpB proteins from these pathogens is only ~40 %. It is likely that ClpB as an important mediator of resistance to oxidative stress could be also a potential target for the host immune response during leptospiral infections in mammals. Therefore, we decided to investigate an immunogenic potential of this chaperone in *Leptospira.* The use of the *E. coli* expression system allowed us to produce the recombinant ClpB_Li_ protein and to assess its immune reactivity with sera collected from *Leptospira*-infected animals and the uninfected healthy controls. Our results show that ClpB is immunogenic during leptospiral infections because it was recognized by sera collected from experimentally infected animals (see Figs. 4 and 5). Thus, among the antibodies raised against leptospiral proteins, there were specific antibodies against ClpB_Li_. This is the first study where ClpB from pathogenic *Leptospira* species was evaluated for its ability to elicit immune responses in animals. Moreover, our results suggest that ClpB_Li_ could be considered as a potential antigen candidate for a diagnostic test. We postulate that the presence of species-specific domains (e.g. ND or MD, see Fig. 1) in the antigen could minimize a cross-reactivity of antibodies with ClpB from different bacteria. Further prospective studies are needed to assess the ClpB_Li_’s predictive value in leptospirosis diagnostics. In addition, the presence of ClpB_Li_ in the infected hamster kidney tissues (see Fig. 6) demonstrates that the chaperone is produced by pathogen during infection of the host further confirming the involvement of ClpB in the pathogenicity of *Leptospira.*

## Conclusions

Identification of *Leptospira* virulence factors and understanding their properties is crucial for uncovering the diseases mechanisms. This study underlines the potential importance of the chaperone ClpB in leptospiral infections. We believe that our data provide new information, which may lead to a better understanding of the role of ClpB and possibly other stress-response factors in the life cycle of the pathogenic bacterium *L. interrogans.* It is worth noting that since ClpB does not exist in animal cells, it might become a promising target for novel therapies against pathogenic *Leptospira* species. Further studies are needed to determine the biological role of ClpB during leptospiral infection in mammals and its diagnostic or even immunoprotective potential. The recombinant ClpB_Li_ produced in this work will help in further biochemical characterization of this chaperone and the analysis of its function in the pathogen.
